# Supplementary material for: Persistent humoral immune response in youth throughout the COVID-19 pandemic: prospective school-based cohort study
Source: Nat Commun. 2023 Nov 27;14:7764. doi: 10.1038/s41467-023-43330-y (PMC10682435; doi:10.1038/s41467-023-43330-y)
Supplement: Supplementary file 1 — Supplementary Information [file 41467_2023_43330_MOESM1_ESM.pdf]

Persistent humoral immune response in youth throughout the COVID-19 pandemic: prospective school-based cohort study

Authors:

Alessia Raineri [1], Thomas Radtke [1], Sonja Rueegg [1], Sarah R. Haile [1], Dominik Menges [1], Tala Ballouz [1], Agne Ulyte [1], Jan Fehr [1], Daniel L. Cornejo [1], Giuseppe Pantaleo [2], Céline Pellaton [2], Craig Fenwick [2], Milo A. Puhan [1], Susi Kriemler [1]*

* corresponding authorship

[1] Epidemiology, Biostatistics and Prevention Institute (EBPI), University of Zurich, Hirschengraben 84, 8001 Zürich, Zurich, Switzerland

[2] Service of Immunology and Allergy, Lausanne University Hospital (CHUV), University of Lausanne (UNIL), Lausanne, Switzerland

Corresponding author:

Susi Kriemler

Tel: +41 79 217 19 87

susi.kriemlerwiget@uzh.ch

Epidemiology, Biostatistics and Prevention Institute (EBPI), University of Zurich, Hirschengraben 84, 8001 Zürich, Zurich, Switzerland

ORCID: 0000-0002-3384-7940

Author positions, emails, affiliations, and addresses:

Alessia Raineri, alessia.raineri2@uzh.ch, Epidemiology, Biostatistics and Prevention Institute (EBPI), University of Zurich, Hirschengraben 84, 8001 Zürich, Zurich, Switzerland

Thomas Radtke, thomas.radtke@uzh.ch, Epidemiology, Biostatistics and Prevention Institute (EBPI), University of Zurich, Hirschengraben 84, 8001 Zürich, Zurich, Switzerland

Sonja Rueegg, sonja.rueegg@uzh.ch, Epidemiology, Biostatistics and Prevention Institute (EBPI), University of Zurich, Hirschengraben 84, 8001 Zürich, Zurich, Switzerland

Sarah R. Haile, sarah.haile@uzh.ch, Epidemiology, Biostatistics and Prevention Institute (EBPI), University of Zurich, Hirschengraben 84, 8001 Zürich, Zurich, Switzerland

Dominik Menges, dominik.menges@uzh.ch, Epidemiology, Biostatistics and Prevention Institute (EBPI), University of Zurich, Hirschengraben 84, 8001 Zürich, Zurich, Switzerland

Tala Ballouz, tala.ballouz@uzh.ch, Epidemiology, Biostatistics and Prevention Institute (EBPI), University of Zurich, Hirschengraben 84, 8001 Zürich, Zurich, Switzerland

Agne Ulyte, agne.ulyte@uzh.ch, Epidemiology, Biostatistics and Prevention Institute (EBPI), University of Zurich, Hirschengraben 84, 8001 Zürich, Zurich, Switzerland

Jan Fehr, Jan.Fehr@uzh.ch, Epidemiology, Biostatistics and Prevention Institute (EBPI), University of Zurich, Hirschengraben 84, 8001 Zürich, Zurich, Switzerland

Daniel L. Cornejo, daniel.llanascornejo@uzh.ch, Epidemiology, Biostatistics and Prevention Institute (EBPI), University of Zurich, Hirschengraben 84, 8001 Zürich, Zurich, Switzerland

Giuseppe Pantaleo, giuseppe.pantaleo@chuv.ch, Service of Immunology and Allergy, Lausanne University Hospital (CHUV), University of Lausanne (UNIL), Lausanne, Switzerland

Céline Pellaton, celine.pellaton@chuv.ch, Service of Immunology and Allergy, Lausanne University Hospital (CHUV), University of Lausanne (UNIL), Lausanne, Switzerland

Craig Fenwick, craig.fenwick@chuv.ch, Service of Immunology and Allergy, Lausanne University Hospital (CHUV), University of Lausanne (UNIL), Lausanne, Switzerland

Milo A. Puhan, miloalan.puhan@uzh.ch, Epidemiology, Biostatistics and Prevention Institute (EBPI), University of Zurich, Hirschengraben 84, 8001 Zürich, Zurich, Switzerland

Susi Kriemler, susi.kriemlerwiget@uzh.ch, Epidemiology, Biostatistics and Prevention Institute (EBPI), University of Zurich, Hirschengraben 84, 8001 Zürich, Zurich, Switzerland

Supplementary Material

Supplementary Methods: Details on the Bayesian logistic regression model

Methods:

The model formulation we used was based on that published by Stringhini et al 2020 "Seroprevalence of anti-SARS-CoV-2 IgG antibodies in Geneva, Switzerland (SEROCoV-POP): a population-based study" (Stringhini S, et al. Seroprevalence of anti- SARS-CoV-2 IgG antibodies in Geneva, Switzerland (SEROCoV- POP): a population-based study. Lancet 396:313–9 (2020)). Ciao Corona and SEROCoV-POP both belong to Switzerland's Corona Immunitas study program. They provided on github repository HopkinsIDD/serocovpop their entire description and example codes. This model was used in all our prior studies (more details on the model can be found elsewhere (Ulyte, A. et al. Variation in SARS-CoV-2 seroprevalence across districts, schools and classes: baseline measurements from a cohort of primary and secondary school children in Switzerland. BMJ Open 11, e047483 (2021))).

Statistical model:

We aimed to estimate the seroprevalence in a population of schoolchildren (grades 1-9) in the Canton of Zurich (denoted as p^*) (model formulation by Stringhini S, et al. Seroprevalence of anti- SARS-CoV-2 IgG antibodies in Geneva, Switzerland (SEROCoV- POP): a population-based study. Lancet 396:313–9 (2020)). We start by estimating the probability that each person in the survey is seropositive using a Bayesian logistic regression model that accounts for each individual's grade level and sex (together X), school-level clustering α_h, the sensitivity, θ^+, and specificity, θ^-, of the antibody test:

$$x_i \sim Bernoulli(p_i\theta^+ + (1 - p_i) * (1 - \theta^-))$$

$$logit(p_i) = \alpha_h + X_i\beta$$

$$\alpha_h \sim Normal(0, \sigma^2)$$

$$x^+ \sim Binomial(n^+, \theta^+)$$

$$x^- \sim Binomial(n^-, 1 - \theta^-)$$

x_i denotes the antibody test result for the ith person (i=1,…, N). The sensitivity, denoted as θ^+, was determined by using the n^+RT-PCR positive controls from the laboratory validation study,

where $x^+$are the positive tested individuals. The specificity, denoted as θ^-, was determined by using the n^- pre-pandemic negative control samples from the laboratory validation study, where x^- are the positive tested individuals. The test specificity and sensitivity are as follows:

SenASTrIS	Cohort	N tested	N positive	N negative
SARS-CoV-2 PCT Positive	67	63	4	94.0% sensitivity
pre-pandemic healthy	256	2	254	99.2% specificity

The probability of observing a diagnostic positive is a function of the true positive rate and the false negative rate with regards to the true underlying probability of seropositivity p_i for that person. This probability itself is a function of covariates X, which consists of sex and grade level, and their coefficients β, and a random effect for school, α_h ($h = 1, \dots, H$), with variance σ^2. We used naive priors on all parameters to allow for an exploration of the parameter space. The priors on the sensitivity and specificity were flat from 0 to 1, equivalent to $Uniform(0,1)$or $Beta(1,1)$. We used weak $Normal(0,1)$ priors for the logistic regression coefficients β. The prior on the standard deviation of the school effect, σ, was flat from 0 to infinity.

We implemented this model in the Stan (Stan Development Team (2023). "RStan: the R interface to Stan." R package version 2.21.8, https://mc-stan.org/) probabilistic programming language and used the RStan (Comprehensive R Archive Network (CRAN). (2023, January 17). R interface to Stan [R package rstan version 2.21.8]. The Comprehensive R Archive Network. https://cran.r-project.org/web/packages/rstan/index.html) package in R to run the model and analyse outputs. We ran 5,000 iterations (4 chains with 1,500 iterations each with 250 for warm-up) and assessed convergence visually.

Seroprevalence estimates:

For each combination of grade level and sex, we calculated the seropositivity probability for every posterior draw of β and σ. To get the weekly seroprevalence estimate, we computed a weighted average of p, using post-stratification weights that correspond to the demographic distribution of the Canton of Zurich:

$$p = \int_0^1 logit^{-1}(X\beta + \sigma\phi^{-1}(t))dt$$

$$p^* = \sum_{sex}\sum_{grade} \frac{pop_{sex,grade} * pop_{sex,grade}}{pop}$$

In this context, $\phi^{-1}(t)$ represents the quantile function of a standard normal distribution, $pop_{sex,grade}$ which denotes the population within each demographic "cell," and pop denotes the school-age population residing in the Canton of Zurich. Our estimation process involves calculating the average seropositivity probability for the population in each demographic cell by integrating across all potential values of a logit-normal distribution, where the standard deviation is determined by the school-specific random effect σ. (model formulation by Stringhini S, et al. Seroprevalence of anti- SARS-CoV-2 IgG antibodies in Geneva, Switzerland (SEROCoV- POP): a population-based study. Lancet 396:313–9 (2020)).

Supplementary Table 1: Baseline characteristics of the repeated cross-sectional study population at each testing round.

Source data are provided as a Source Data file. a: Predominant variant of concern (VOC) in Switzerland (>50% of circulating variants in Switzerland); b: unique number of children and adolescents tested throughout the entire study period; c: number of children and adolescents tested per round; d: details on chronic conditions can be found in Supplement Table 4; e: grouped into <12 years and ≥12 years, since in Switzerland, adolescents ≥12 years of age could get vaccinated since mid-June 2021 and children between 5 and 11 years of age from January 2022. T1: Jun/Jul 2020; T2: Oct/Nov 2020; T3: Mar/Apr 2021; T4: Nov/Dec 2021; T5: Jun/Jul 2022.

*median (interquartile range)

	T1	T2	T3	T4	T5
**Timeframe of testing**	Jun/Jul 2020	Oct/Nov 2020	Mar/Apr 2021	Nov/Dec 2021	Jun/Jul 2022
**Predominant VOC [a]**	Wildtype	Wildtype	Alpha	Delta	Omicron
**N unique [b]**	4250				
**N tested [c]**	2473	2500	2450	1874	2105
**Age * (years)**	12 (9-14)	12 (9-14)	12 (10-14)	12 (9-13)	12 (10-14)
**Sex (n, % male)**	1197 (48%)	1211 (48%)	1165 (48%)	883 (47%)	990 (47%)
**Age group**					
**<12yrs**	1450 (59%)	1298 (52%)	1144 (47%)	945 (50%)	1011 (48%)
**≥12yrs**	1023 (41%)	1202 (48%)	1306 (53%)	929 (50%)	1094 (52%)
**Presence of any chronic condition [d]**	546 (24%)	551 (24%)	536 (24%)	375 (23%)	412 (23%)

**Vaccinated** [e]					
**Overall**	0	0	0	475/1874 (25%)	913/2105 (43%)
**<12yr**				0	283/1011 (28%)
**≥12yr**				475/929 (51%)	630/1094 (58%)
**Questionnaires completed**	2211 (89%)	2030 (81%)	1897 (77%)	1461 (78%)	1499 (71%)
**Participation rate within a class * (%)**	47% (27%-61%)	47% (29%-61%)	50% (35%-63%)	38% (24%-50%)	36% (23%-50%)

Supplementary Table 2: Overall seroprevalence and seroprevalence split by <12 and ≥12-year-old children and adolescents.

This table illustrates the overall seroprevalence and seroprevalence divided according to age (<12 years and ≥ 12years) for all five testing rounds. *indicates the proportion of vaccinated children and adolescents per testing round. Source data are provided as a Source Data file.

At T4, 25.3% of all children and adolescents were vaccinated (all of whom were ≥12yrs old), and at T5 43.4% (28% of children <12 and 58% of adolescents ≥12yrs old). When stratifying according to age, there was a larger increase in seroprevalence between T4 and T5 in children below the age of 12 (28.4% [95% CrI 24.2-33.2%] to 95.7% [95% CrI 93.5-97.4%], respectively) compared to adolescents of 12 years or more (69.5% [95% CrI 63.8-75.7%] to 98.4% [95% CrI 97.3-99.1%], respectively).

	Timeframe of testing	*T1*<br>*Jun/July 2020*	*T2*<br>*Nov/Dec 2020*	*T3*<br>*Mar/Apr 2021*	*T4*<br>*Oct/Nov 2021*	*T5*<br>*Jun/Jul 2022*
All	**Seroprevalence:**					
	Unvaccinated	3.0%<br>(1.3% - 4.5%)	5.6%<br>(3.6% - 7.6%)	18.4%<br>(15.2% - 21.9%)	31.3%<br>(27.5% - 35.9%)	95.7%<br>(93.0% - 97.7%)
	Vaccinated ± infected	-	-	-	46.5%<br>(42.5% - 51.3%)	96.9%<br>(95.3% - 98.1%)
	% vaccinated *	-	-	-	25.3%	43.4%
<12yrs	**Seroprevalence:**					
	Unvaccinated	3.0%<br>(1.4% - 4.6%)	5.6%<br>(3.5% - 7.9%)	19.7%<br>(16.0% - 23.9%)	28.4%<br>(24.2% - 33.2%)	95.0%<br>(91.9% - 97.4%)

	Vaccinated ± infected	-	-	-	-	95.7% (93.5% - 97.4%)
	% vaccinated *	-	-	-	0	28.0 %
≥12yrs	**Seroprevalence:**					
	Unvaccinated	2.9% (0.9% -4.9%)	5.6% (3.2% - 7.9%)	16.8% (13.6% - 20.0%)	40.3% (34.2% - 47.0%)	97.1% (94.5% - 98.7%)
	Vaccinated ± infected	-	-	-	69.5% (63.8% - 75.7%)	98.4% (97.3% - 99.1%)
	% vaccinated *	-	-	-	51.1%	57.6%

Supplementary Table 3: Clinical details of children and adolescents with any hospital stay during the study period.

Characteristics of children and adolescents that have been hospitalised over the entire study period.

* Mean fluorescence intensity (MFI) values above the threshold of ≥6 indicates seropositive anti-spike IgG response.

Participant	*Time of reported symptoms*	*Age*	*Sex*	*Chronic*	*Symptoms reported*	*Anti-spike IgG antibodies (MFI ratio) **	*Days of hospital stays*	*Phone call with parents / Comments*
1	T4: Nov/Dec 2021	≥12	m	-	Fever, sore throat, headache, muscle and joint pain, tiredness or fatigue	T1: 3.6<br>T2: 3.6<br>T3: 4.3<br>T4: 62.4<br>T5: 112.2	<24 hours	-
2	T1: Jun/Jul 2020	<12	m	Asthma, Allergies	Fever, cough, runny or stuffy nose, sneeze, sore throat, headache, muscle and joint pain, tiredness or fatigue, loss of appetite, nausea/vomiting, upset stomach, loss of smell and taste	T1: 8<br>T2: 3<br>T3: 0.9<br>T4: 0.5<br>T5: 7	<24 hours	Was taken to the hospital because the child hit his head.
3	T5: Jun/Jul 2022	≥12	f	hay fever, neurodermitis/eczema, joint disease	Stomach ache, dizziness	T1: -<br>T2: -<br>T3: -<br>T4: 0.1<br>T5: 131.5	<24 hours	Only short emergency room visit

Supplementary Table 4: Chronic conditions in children and adolescents.

Chronic disease assessed via questionnaires. In this table you find all chronic conditions reported by the children and adolescents or their parents.

**Chronic conditions**
Asthma
hay fever
celiac disease
lactose intolerance
allergies (other than hay fever)
neurodermitis
diabetes mellitus
inflammatory bowel disease
hypertension
arthritis
other chronic diseases potentially affecting the immune response (Neutropenia, PFAPA-Syndrome, renal failure, cystic fibrosis, bronchitis)

Supplementary Table 5: Anti-spike IgG mean fluorescence intensity (MFI) ratios and neutralising activity (IC50) at T5.

(a) This table provides detailed anti-spike IgG titres (mean fluorescence intensity (MFI) ratio, seropositive values defined by a cutoff of ≥6) and neutralising activity (measured by the half maximal inhibitory concentration 50 (IC50), with positive values defined by a cutoff of 50 or higher) in children and adolescents from the longitudinal cohort. We divided individuals based on their serology and exposure status at T5 in Hybrid, Vaccinated and Infected. **(b)** This table provides the MFI ratio converted to WHO units per millilitre (U/ml) for Roche Elecsys anti-spike IgG. We converted the MFI values to WHO U/ml, by using the Elecsys Anti-SARS-CoV2 immunoassay developed by Roche, for the purpose of interpretation. The Department of Clinical Immunology & Allergy of the University Hospital of Lausanne used population based samples to provide the conversion formula of Roche $anti-spike\ IgG = 10\text{^}(-0.6108069 + 2.0072882 \times log10(MFI + 1))$. Source data are provided as a Source Data file.

MFI: Mean Fluorescence intensity; IC50: half maximal inhibitory concentration 50; IQR: Inter Quartile Range; CI: Confidence Interval

(a)

N	*T5 Group*	*Anti-spike IgG titres (MFI Ratio) Median and IQR*	*Anti-Wildtype neutralising activity (IC50) Median (IQR)*	*Anti-Delta neutralising activity (IC50) Median (IQR)*	*Anti-Omicron BA.1 neutralising activity (IC50) Median (IQR)*	*% of Individuals above threshold for Anti-Wildtype (95% CI)*	*% of Individuals above threshold for Anti-Delta (95% CI)*	*% of Individuals above threshold for Anti-Omicron BA.1 (95% CI)*
180	Hybrid	136.2 (121.9-154.3)	591.2 (355.4-1055.2)	316.9 (189.1-574.1)	220 (147.7-382.9)	98.3 (95.2-99.4)	96.1 (92.2-98.1)	98.9 (96.0-99.7)
158	Vaccinated	127.6	312.8 (173-689.4)	158.4 (88.9-337.4)	133.2 (60.5-214.4)	96.2 (92.0-98.2)	86.7 (80.5-91.1)	81.6 (74.9-86.9)

		(114.1-151)						
353	Infected	54.8 (22.8-89.8)	59.2 (16.7-125.9)	42.5 (11.9-88.3)	69.5 (34.2-127.4)	54.4 (49.2-59.5)	45.6 (40.5-50.8)	64.9 (59.8-69.7)

(b)

N	***T5 Group***	***Anti-spike IgG titres (MFI Ratio) Median and IQR***	***Anti-Wildtype neutralising activity (IC50) Median (IQR)***	***Anti-Delta neutralising activity (IC50) Median (IQR)***	***Anti-Omicron BA.1 neutralising activity (IC50) Median (IQR)***	***% of Individuals above threshold for Anti-Wildtype (95% CI)***	***% of Individuals above threshold for Anti-Delta (95% CI)***	***% of Individuals above threshold for Anti-Omicron BA.1 (95% CI)***
180	Hybrid	4784.1 (3829.8-6130.6)	591.2 (355.4-1055.2)	316.9 (189.1-574.1)	220 (147.7-382.9)	98.3 (95.2-99.4)	96.1 (92.2-98.1)	98.9 (96.0-99.7)
158	Vaccinated	4198.2 (3360.2-5874.1)	312.8 (173-689.4)	158.4 (88.9-337.4)	133.2 (60.5-214.4)	96.2 (92.0-98.2)	86.7 (80.5-91.1)	81.6 (74.9-86.9)
353	Infected	785.6 (142-2087.5)	59.2 (16.7-125.9)	42.5 (11.9-88.3)	69.5 (34.2-127.4)	54.4 (49.2-59.5)	45.6 (40.5-50.8)	64.9 (59.8-69.7)

Supplementary Table 6: Anti-spike IgG mean fluorescence intensity (MFI) ratios between T4 (Nov/Dec 2021) and T5 (Jun/Jul 2022).

(a) This table provides the anti-spike IgG mean fluorescence intensity (MFI) ratio (median and interquartile range) shown in the boxplot (Figure 2) for all different group combinations. **(b)** This table provides the MFI ratio converted to WHO units per millilitre (U/ml) shown in the boxplot (Supplementary Figure 5). Source data are provided as a Source Data file.

MFI: Mean Fluorescence intensity; IQR: Inter Quartile Range

(a)

N	*T4 Group*	*T5 Group*	*T4: Anti-spike IgG titres (MFI Ratio) Median and IQR*	*T5: Anti-spike IgG titres (MFI Ratio) Median and IQR*
38	T4 Hybrid	T5 Hybrid	107.2 (88.8-113.1)	132.8 (121.6-145.9)
60	T4 Vaccinated	T5 Hybrid	103 (80.6-119.8)	135.1 (121.6-155.3)
84	T4 Vaccinated	T5 Vaccinated	111.2 (89.3-122.6)	128.4 (118.7-155.1)
33	T4 Infected	T5 Hybrid	35.8 (24.8-48.5)	150.5 (130.4-158.4)
143	T4 Infected	T5 Infected	36.5 (24.9-50.2)	86.4 (64.2-117.8)
49	T4 Negative	T5 Hybrid	1 (1-1)	136.2 (119.1-149.2)
74	T4 Negative	T5 Vaccinated	1 (1-1)	125.4 (94.3-141.4)
210	T4 Negative	T5 Infected	1 (1-1.2)	27.9 (15.9-56.4)

(b)

N	*T4 Group*	*T5 Group*	*T4: Anti-spike IgG WHO U/ml Median and IQR*	*T5: Anti-spike IgG WHO U/ml Median and IQR*
38	T4 Hybrid	T5 Hybrid	2968.1 (2044.4-3303.3)	4542.3 (3812.9-5479.4)
60	T4 Vaccinated	T5 Hybrid	2744 (1685.8-3706.2)	4700.7 (3815.7-6212.1)
84	T4 Vaccinated	T5 Vaccinated	3192.4 (2064.5-3875.3)	4250.6 (3636.7-6190.2)
33	T4 Infected	T5 Hybrid	340.6 (167-617.7)	5833.2 (4383.5-6459.8)
143	T4 Infected	T5 Infected	353.8 (167.7-662.3)	1933.6 (1075.4-3577.5)
49	T4 Negative	T5 Hybrid	1 (1-1)	4780.6 (3659.6-5733.2)
74	T4 Negative	T5 Vaccinated	1 (1-1)	4058.9 (2303.2-5151.2)
210	T4 Negative	T5 Infected	1 (1-1.2)	209 (71.6-832.2)

Supplementary Table 7: Anti-spike IgG mean fluorescence intensity (MFI) ratios between T4 (Nov/Dec 2021) and T5 (Jun/Jul 2022) stratified by age (<12 and ≥12 years).

These tables provide the anti-spike IgG mean fluorescence intensity (MFI) ratio (median and interquartile range) shown in the boxplot (Supplementary Figure 6) for all different group combinations stratified by age. **(a)** For the age group <12 years of age. **(b)** For the age group ≥12 years of age. **(c)** For the age group <12 years with MFI ratio converted to WHO units per millilitre (U/ml). **(d)** For the age group ≥12 years of age with MFI ratio converted to WHO U/ml. Source data are provided as a Source Data file.

MFI: Mean Fluorescence intensity; IQR: Inter Quartile Range

(a)

N	*T4 Group*	*T5 Group*	*T4: Anti-spike IgG titres (MFI ratio) Median and IQR*	*T5: Anti-spike IgG titres (MFI ratio) Median and IQR*
22	T4 Infected	T5 Hybrid	31 (21.6-47.4)	147.1 (131.6-157.9)
91	T4 Infected	T5 Infected	38.6 (28-51.6)	85 (64.4-118.8)
42	T4 Negative	T5 Hybrid	1 (1-1)	136.8 (119.1-150.8)
57	T4 Negative	T5 Vaccinated	1 (1-1)	124 (89.5-138.7)
140	T4 Negative	T5 Infected	1 (1-1.2)	34.7 (18.4-64.3)

(b)

N	*T4 Group*	*T5 Group*	*T4: Anti-spike IgG titres (MFI ratio) Median and IQR*	*T5: Anti-spike IgG titres (MFI ratio) Median and IQR*
38	T4 Hybrid	T5 Hybrid	107.2 (88.8-113.1)	132.8 (121.6-145.9)
60	T4 Vaccinated	T5 Hybrid	103 (80.6-119.8)	135.1 (121.6-155.3)
84	T4 Vaccinated	T5 Vaccinated	111.2 (89.3-122.6)	128.4 (118.7-155.1)
11	T4 Infected	T5 Hybrid	40.1 (34-55.4)	152 (131.4-161.4)
52	T4 Infected	T5 Infected	35.2 (19.4-47.8)	90 (64.1-117.2)

7	T4 Negative	T5 Hybrid	1 (1-1.1)	130.6 (107.4-134.6)
17	T4 Negative	T5 Vaccinated	1 (1-2.2)	135.7 (114-149.4)
70	T4 Negative	T5 Infected	1 (1-1.2)	20.1 (14.3-42.5)

(c)

N	***T4 Group***	***T5 Group***	***T4: Anti-spike IgG WHO U/ml Median and IQR***	***T5: Anti-spike IgG WHO U/ml Median and IQR***
22	T4 Infected	T5 Hybrid	258.2 (127.8-590.8)	5571.2 (4463.6-6419.4)
91	T4 Infected	T5 Infected	394.7 (211.9-697.8)	1871.9 (1082.1-3638.3)
42	T4 Negative	T5 Hybrid	1 (1-1)	4822.7 (3659.6-5854.7)
57	T4 Negative	T5 Vaccinated	1 (1-1)	3965.5 (2073.7-4957)
140	T4 Negative	T5 Infected	1 (1-1.2)	320.6 (94-1077.1)

(d)

N	***T4 Group***	***T5 Group***	***T4: Anti-spike IgG WHO U/ml Median and IQR***	***T5: Anti-spike IgG WHO U/ml Median and IQR***
38	T4 Hybrid	T5 Hybrid	2968.1 (2044.4-3303.3)	4542.3 (3812.9-5479.4)
60	T4 Vaccinated	T5 Hybrid	2744 (1685.8-3706.2)	4700.7 (3815.7-6212.1)
84	T4 Vaccinated	T5 Vaccinated	3192.4 (2064.5-3875.3)	4250.6 (3636.7-6190.2)
11	T4 Infected	T5 Hybrid	425.2 (308.1-835.8)	5949.7 (4455.2-6705.3)
52	T4 Infected	T5 Infected	329.8 (103.7-600.9)	2094.5 (1069.7-3544.3)
7	T4 Negative	T5 Hybrid	1 (1-1.1)	4396.9 (3038.4-4666.6)
17	T4 Negative	T5 Vaccinated	1 (1-2.5)	4745.7 (3354.3-5748.5)
70	T4 Negative	T5 Infected	1 (1-1.2)	111.5 (58.7-476.1)

Supplementary Table 8: Proportion of children and adolescents with reinfection between T4 (Nov/Dec 2021) and T5 (Jun/Jul 2022).

(a) This table shows the proportion of children and adolescents with first SARS-CoV-2 infection before T4 (Nov/Dec 2021) and reinfection between T4 (Nov/Dec 2021) and T5 (Jun/Jul 2022). We divided those children and adolescents into infected only or vaccinated, irrespective of infection prior to T4. We divided children and adolescents into infected only, those with older vaccination (last vaccination prior to T4) and those with recent vaccination (last vaccination between T4 and T5), irrespective of infection prior to T4. In these three groups we calculated the proportion of children and adolescents experiencing reinfection by looking at the presence of a newly anti-nucleocapsid IgG antibody as well as an arbitrary cutoff of a ≥25% increase and/or decrease of anti-spike IgG antibody. **(b)** This table shows the sensitivity analysis using two different thresholds (15% and 35%). Source data are provided as a Source Data file.

* Anti-nucleocapsid IgG positivity (+: seropositive; -: seronegative)

[a] shows the children and adolescents with the last vaccination between T4 and T5, irrespective of infection prior to T4.

[b] shows the children and adolescents with last vaccination prior to T4, irrespective of infection prior to T4.

(a)

	**Unvaccinated N=314**		**Recent vaccination [a] N=221**		**Older vaccination [b] N=206**	
**Anti-spike IgG titre increase or decrease between T4 and T5**	Anti-N IgG +*	Anti-N IgG -*	Anti-N IgG +*	Anti-N IgG -*	Anti-N IgG +*	Anti-N IgG -*
**≥25% increase**	231/314 (73.6%)	31/314 (9.9%)	57/221 (25.5%)	101/221 (45.7%)	58/205 (28.3%)	52/205 (25.4%)
**<25% increase**	21/314 (6.7 %)	31/314 (9.9%)	24/221 (10.9%)	39/221 (17.6%)	44/205 (21.5%)	51/205 (24.9%)

(b)

	**Unvaccinated N=314**		**Recent vaccination [a] N=221**		**Older vaccination [b] N=205**	
**Anti-spike IgG titre increase or decrease between T4 and T5**	Anti-N IgG +*	Anti-N IgG -*	Anti-N IgG +*	Anti-N IgG -*	Anti-N IgG +*	Anti-N IgG -*
**≥15% increase**	236/314 (75.2%)	38/314 (12.1%)	60/221 (27.1%)	108/221 (48.9%)	67/205 (32.7%)	63/205 (30.7%)
**<15% increase**	16/314 (5.1%)	24/314 (7.6%)	21/221 (9.5%)	32/221 (14.5%)	35/205 (17.1%)	40/205 (19.5%)
**≥35% increase**	229/314 (72.9%)	25/314 (8.0%)	51/221 (23.1%)	96/221 (43.4%)	49/205 (23.9%)	41/205 (20.0%)
**<35% increase**	23/314 (7.3%)	37/314 (11.8%)	30/221 (13.6%)	44/221 (19.9%)	53/205 (25.9%)	62/205 (30.2%)

Supplementary Table 9: Neutralising activity between T4 (Nov/Dec 2021) and T5 (Jun/Jul 2022).

This table shows the development of neutralising antibodies against different SARS-CoV-2 variants between T4 (Nov/Dec 2021) and T5 (Jun/Jul 2022) (see Figure 3). We again separated participants according to their serology and exposure status at T4 and exposure status (i.e., negative, infected, vaccinated, or hybrid) and evaluated neutralising activity against Wildtype, Delta, and Omicron BA.1. We calculated medians (inter quartile ranges (IQR)) and proportion of children and adolescents with neutralising activity, measured by the half maximal inhibitory concentration (IC50) with positive or negative results defined by a cutoff value of 50 or higher. Source data are provided as a Source Data file.

IC50: half maximal inhibitory concentration 50; IQR: Inter Quartile Range; CI: Confidence Interval

N	*Variant of concern*	*T4 Group*	*T5 Group*	*T4: Neutralising activity (IC50) Median (IQR)*	*T5: Neutralising activity (IC50) Median (IQR)*	*T4: % of Individuals above threshold (95% CI)*	*T5: % of Individuals above threshold (95% CI)*
38	Wildtype	T4 Hybrid	T5 Hybrid	913.9 (476.9-2287.4)	672.5 (339.1-1015.2)	100 (90.8-100)	100 (90.8-100)
60	Wildtype	T4 Vaccinated	T5 Hybrid	568.5 (324.4-780.8)	1082.9 (639.9-2083.3)	100 (94-100)	100 (94-100)
84	Wildtype	T4 Vaccinated	T5 Vaccinated	405.2 (254.9-569.3)	578.1 (319.1-929.3)	98.8 (93.6-99.8)	100 (95.6-100)
33	Wildtype	T4 Infected	T5 Hybrid	51.9 (34-82.2)	505.8 (372.9-687.8)	51.5 (35.2-67.5)	100 (89.6-100)
143	Wildtype	T4 Infected	T5 Infected	59.7 (35.8-89.4)	125.9 (81.6-206.3)	58 (49.8-65.8)	90.9 (85.1-94.6)
49	Wildtype	T4 Negative	T5 Hybrid	0 (0-0)	354 (205.4-494.1)	0 (0-7.3)	93.9 (83.5-97.9)
74	Wildtype	T4 Negative	T5 Vaccinated	0 (0-0)	207.3 (119-281.8)	0 (0-4.9)	91.9 (83.4-96.2)
210	Wildtype	T4 Negative	T5 Infected	0 (0-0)	21.5 (11.1-59.2)	0 (0-1.8)	29.5 (23.8-36)
38	Delta	T4 Hybrid	T5 Hybrid	395.3 (219.4-919.9)	318.6 (187.4-547.7)	97.4 (86.5-99.5)	100 (90.8-100)

60	Delta	T4 Vaccinated	T5 Hybrid	211.3 (129.2-337.5)	610.2 (377.7-1143.4)	100 (94-100)	100 (94-100)
84	Delta	T4 Vaccinated	T5 Vaccinated	170.5 (118.4-240.4)	256.9 (153-490.5)	96.4 (90-98.8)	94 (86.8-97.4)
33	Delta	T4 Infected	T5 Hybrid	31.7 (21.2-49.7)	249 (187.4-324.7)	24.2 (12.8-41)	100 (89.6-100)
143	Delta	T4 Infected	T5 Infected	33.9 (22-47.7)	88.3 (56.6-141.3)	23.1 (16.9-30.6)	79.7 (72.4-85.5)
49	Delta	T4 Negative	T5 Hybrid	0 (0-0)	189.8 (136.1-308.7)	0 (0-7.3)	85.7 (73.3-92.9)
74	Delta	T4 Negative	T5 Vaccinated	0 (0-0)	112.8 (55.2-150.1)	0 (0-4.9)	78.4 (67.7-86.2)
210	Delta	T4 Negative	T5 Infected	0 (0-0)	16 (8.3-45.8)	0 (0-1.8)	22.4 (17.3-28.5)
38	Omicron BA.1	T4 Hybrid	T5 Hybrid	191 (105.2-630.5)	203.3 (124.7-288.8)	92.1 (79.2-97.3)	100 (90.8-100)
60	Omicron BA.1	T4 Vaccinated	T5 Hybrid	95 (60.5-143.9)	421.9 (284.1-656.5)	81.7 (70.1-89.4)	100 (94-100)
84	Omicron BA.1	T4 Vaccinated	T5 Vaccinated	70.1 (49.8-95)	191.7 (79.5-329.9)	75 (64.8-83)	84.5 (75.3-90.7)
33	Omicron BA.1	T4 Infected	T5 Hybrid	0 (0-15.8)	166.2 (124.8-206.1)	0 (0-10.4)	100 (89.6-100)
143	Omicron BA.1	T4 Infected	T5 Infected	0 (0-13)	85.2 (54.2-126.8)	1.4 (0.4-5)	78.3 (70.9-84.3)
49	Omicron BA.1	T4 Negative	T5 Hybrid	0 (0-0)	176.9 (125.9-252.1)	0 (0-7.3)	95.9 (86.3-98.9)
74	Omicron BA.1	T4 Negative	T5 Vaccinated	0 (0-0)	89.6 (54.4-158.5)	0 (0-4.9)	78.4 (67.7-86.2)
210	Omicron BA.1	T4 Negative	T5 Infected	0 (0-0)	57.3 (25.1-129.5)	0 (0-1.8)	55.7 (49-62.3)

Supplementary Table 10: Neutralising activity between T4 (Nov/Dec 2021) and T5 (Jun/Jul 2022) stratified by age (<12 and ≥12 years).

This table shows the development of neutralising antibodies against different SARS-CoV-2 variants between T4 (Nov/Dec 2021) and T5 (Jun/Jul 2022), stratified by age (Supplementary Figure 7). **(a)** For the age group <12 years of age. **(b)** For the age group ≥12 years of age. Source data are provided as a Source Data file.

IC50: half maximal inhibitory concentration 50; IQR: Inter Quartile Range; CI: Confidence Interval

(a)

N	*Variant of concern*	*T4 Group*	*T5 Group*	*T4: Neutralising activity (IC50) Median (IQR)*	*T5: Neutralising activity (IC50) Median (IQR)*	*T4: % of Individuals above threshold (95% CI)*	*T5: % of Individuals above threshold (95% CI)*
22	Wildtype	T4 Infected	T5 Hybrid	48.7 (24.7-69.3)	489.7 (385.8-574.7)	50 (30.7%, 69.3%)	100 (85.1%, 100%)
91	Wildtype	T4 Infected	T5 Infected	60.2 (38.2-98.4)	126.6 (79.9-202.2)	60.4 (50.2%, 69.9%)	91.2 (83.6%, 95.5%)
42	Wildtype	T4 Negative	T5 Hybrid	0 (0-0)	332.7 (204.5-484.4)	0 (0%, 8.4%)	95.2 (84.2%, 98.7%)
57	Wildtype	T4 Negative	T5 Vaccinated	0 (0-0)	191 (106.6-265.5)	0 (0%, 6.3%)	91.2 (81.1%, 96.2%)
140	Wildtype	T4 Negative	T5 Infected	0 (0-0)	26.3 (13.8-66.5)	0 (0%, 2.7%)	32.9 (25.6%, 41%)
22	Delta	T4 Infected	T5 Hybrid	32 (16.3-44.6)	242.3 (200-323.9)	13.6 (4.7%, 33.3%)	100 (85.1%, 100%)
91	Delta	T4 Infected	T5 Infected	37.9 (23.4-50.5)	91.6 (50.3-147.6)	26.4 (18.4%, 36.3%)	75.8 (66.1%, 83.5%)
42	Delta	T4 Negative	T5 Hybrid	0 (0-0)	181.3 (134.9-283.8)	0 (0%, 8.4%)	85.7 (72.2%, 93.3%)

57	Delta	T4 Negative	T5 Vaccinated	0 (0-0)	105.3 (53.2-138.1)	0 (0%, 6.3%)	75.4 (62.9%, 84.8%)
140	Delta	T4 Negative	T5 Infected	0 (0-0)	18.2 (9.5-51.8)	0 (0%, 2.7%)	27.1 (20.5%, 35%)
22	Omicron BA.1	T4 Infected	T5 Hybrid	0 (0-0)	164.9 (126.2-197.1)	0 (0%, 14.9%)	100 (85.1%, 100%)
91	Omicron BA.1	T4 Infected	T5 Infected	0 (0-14.5)	79.5 (49.8-124.7)	2.2 (0.6%, 7.7%)	74.7 (64.9%, 82.5%)
42	Omicron BA.1	T4 Negative	T5 Hybrid	0 (0-0)	172.4 (125.8-249.6)	0 (0%, 8.4%)	95.2 (84.2%, 98.7%)
57	Omicron BA.1	T4 Negative	T5 Vaccinated	0 (0-0)	88.8 (54.4-147.5)	0 (0%, 6.3%)	78.9 (66.7%, 87.5%)
140	Omicron BA.1	T4 Negative	T5 Infected	0 (0-0)	68.3 (28.7-141.8)	0 (0%, 2.7%)	60.7 (52.4%, 68.4%)

(b)

N	***Variant of concern***	***T4: Group***	***T5: Group***	***T4: Median (IQR)***	***T5: Median (IQR)***	***T4: % of Individuals above threshold (%)***	***T5: % of Individuals above threshold (%)***
38	Wildtype	T4 Hybrid	T5 Hybrid	913.9 (476.9-2287.4)	672.5 (339.1-1015.2)	100 (90.8%, 100%)	100 (90.8%, 100%)
60	Wildtype	T4 Vaccinated	T5 Hybrid	568.5 (324.4-780.8)	1082.9 (639.9-2083.3)	100 (94%, 100%)	100 (94%, 100%)
84	Wildtype	T4 Vaccinated	T5 Vaccinated	405.2 (254.9-569.3)	578.1 (319.1-929.3)	98.8 (93.6%, 99.8%)	100 (95.6%, 100%)
11	Wildtype	T4 Infected	T5 Hybrid	59.2 (41.2-109.2)	552.8 (417.5-829.1)	54.5 (28%, 78.7%)	100 (74.1%, 100%)
52	Wildtype	T4 Infected	T5 Infected	57.2 (32.1-70.4)	125.4 (82.9-207.8)	53.8 (40.5%, 66.7%)	90.4 (79.4%, 95.8%)
7	Wildtype	T4 Negative	T5 Hybrid	0 (0-0)	447 (265.2-562.5)	0 (0%, 35.4%)	85.7 (48.7%, 97.4%)
17	Wildtype	T4 Negative	T5 Vaccinated	0 (0-0)	298.1 (167.4-655.3)	0 (0%, 18.4%)	94.1 (73%, 99%)
70	Wildtype	T4 Negative	T5 Infected	0 (0-0)	16.8 (8.4-36.4)	0 (0%, 5.2%)	22.9 (14.6%, 34%)
38	Delta	T4 Hybrid	T5 Hybrid	395.3 (219.4-919.9)	318.6 (187.4-547.7)	97.4 (86.5%, 99.5%)	100 (90.8%, 100%)

60	Delta	T4 Vaccinated	T5 Hybrid	211.3 (129.2-337.5)	610.2 (377.7-1143.4)	100 (94%, 100%)	100 (94%, 100%)
84	Delta	T4 Vaccinated	T5 Vaccinated	170.5 (118.4-240.4)	256.9 (153-490.5)	96.4 (90%, 98.8%)	94 (86.8%, 97.4%)
11	Delta	T4 Infected	T5 Hybrid	29.8 (21.4-58.4)	276.6 (176.4-385.9)	45.5 (21.3%, 72%)	100 (74.1%, 100%)
52	Delta	T4 Infected	T5 Infected	30.5 (19.7-42.6)	83.7 (62-138.7)	17.3 (9.4%, 29.7%)	86.5 (74.7%, 93.3%)
7	Delta	T4 Negative	T5 Hybrid	0 (0-0)	254.4 (210.2-314.1)	0 (0%, 35.4%)	85.7 (48.7%, 97.4%)
17	Delta	T4 Negative	T5 Vaccinated	0 (0-0)	149.9 (97-398.9)	0 (0%, 18.4%)	88.2 (65.7%, 96.7%)
70	Delta	T4 Negative	T5 Infected	0 (0-0)	11.3 (5.7-21.9)	0 (0%, 5.2%)	12.9 (6.9%, 22.7%)
38	Omicron BA.1	T4 Hybrid	T5 Hybrid	191 (105.2-630.5)	203.3 (124.7-288.8)	92.1 (79.2%, 97.3%)	100 (90.8%, 100%)
60	Omicron BA.1	T4 Vaccinated	T5 Hybrid	95 (60.5-143.9)	421.9 (284.1-656.5)	81.7 (70.1%, 89.4%)	100 (94%, 100%)
84	Omicron BA.1	T4 Vaccinated	T5 Vaccinated	70.1 (49.8-95)	191.7 (79.5-329.9)	75 (64.8%, 83%)	84.5 (75.3%, 90.7%)
11	Omicron BA.1	T4 Infected	T5 Hybrid	0 (0-26)	182.9 (126.8-242.7)	0 (0%, 25.9%)	100 (74.1%, 100%)
52	Omicron BA.1	T4 Infected	T5 Infected	0 (0-8.2)	87.9 (62.7-126.9)	0 (0%, 6.9%)	84.6 (72.5%, 92%)
7	Omicron BA.1	T4 Negative	T5 Hybrid	0 (0-0)	243 (186.2-313.5)	0 (0%, 35.4%)	100 (64.6%, 100%)
17	Omicron BA.1	T4 Negative	T5 Vaccinated	0 (0-0)	138.6 (62.6-189.4)	0 (0%, 18.4%)	76.5 (52.7%, 90.4%)
70	Omicron BA.1	T4 Negative	T5 Infected	0 (0-0)	47 (22-91.3)	0 (0%, 5.2%)	45.7 (34.6%, 57.3%)

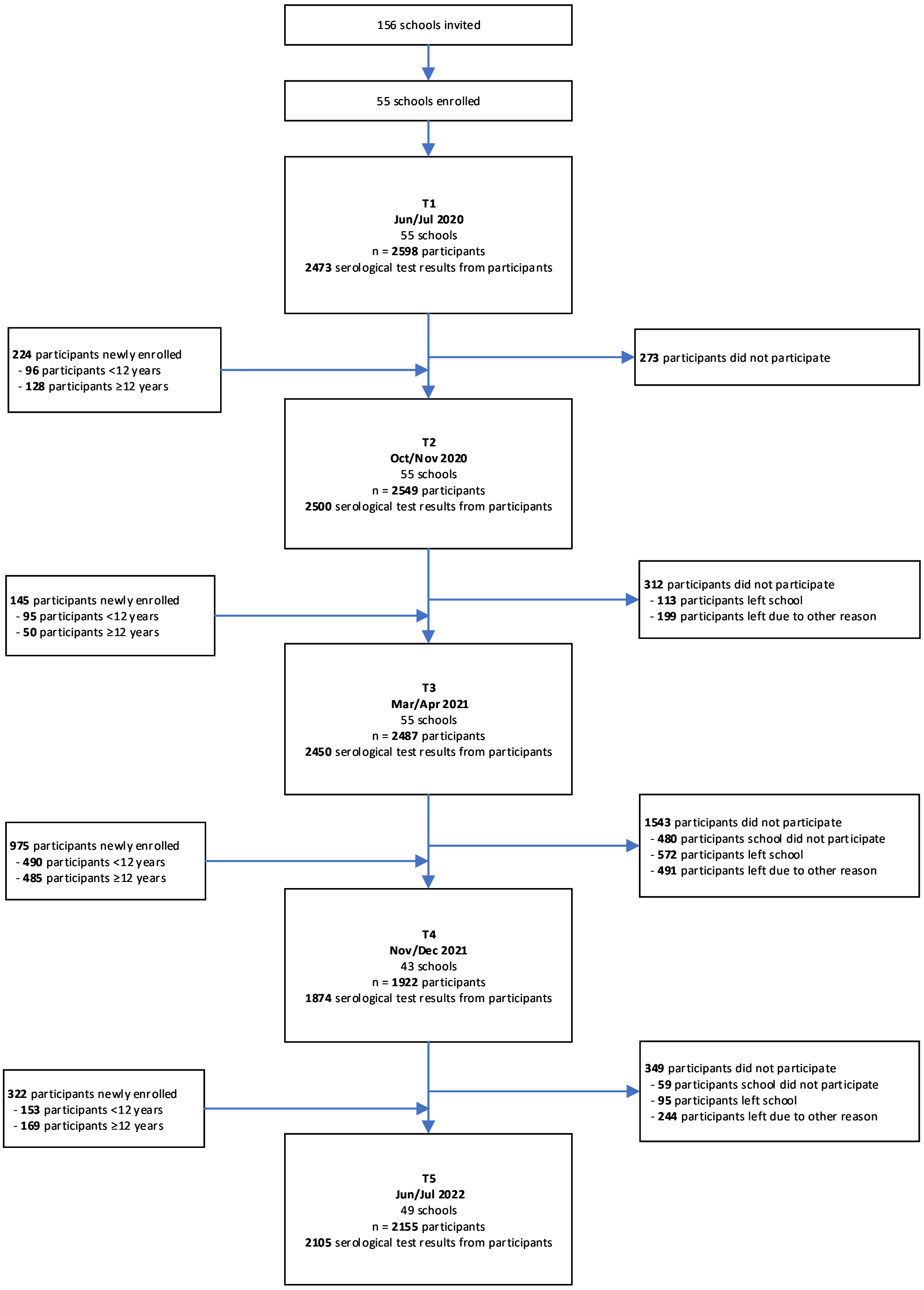

Supplementary Figure 1: Flowchart of participants.

This figure shows the flowchart of participants over the five testing rounds from June 2020 to July 2022. Newly enrolled children and adolescents did not participate in previous rounds.

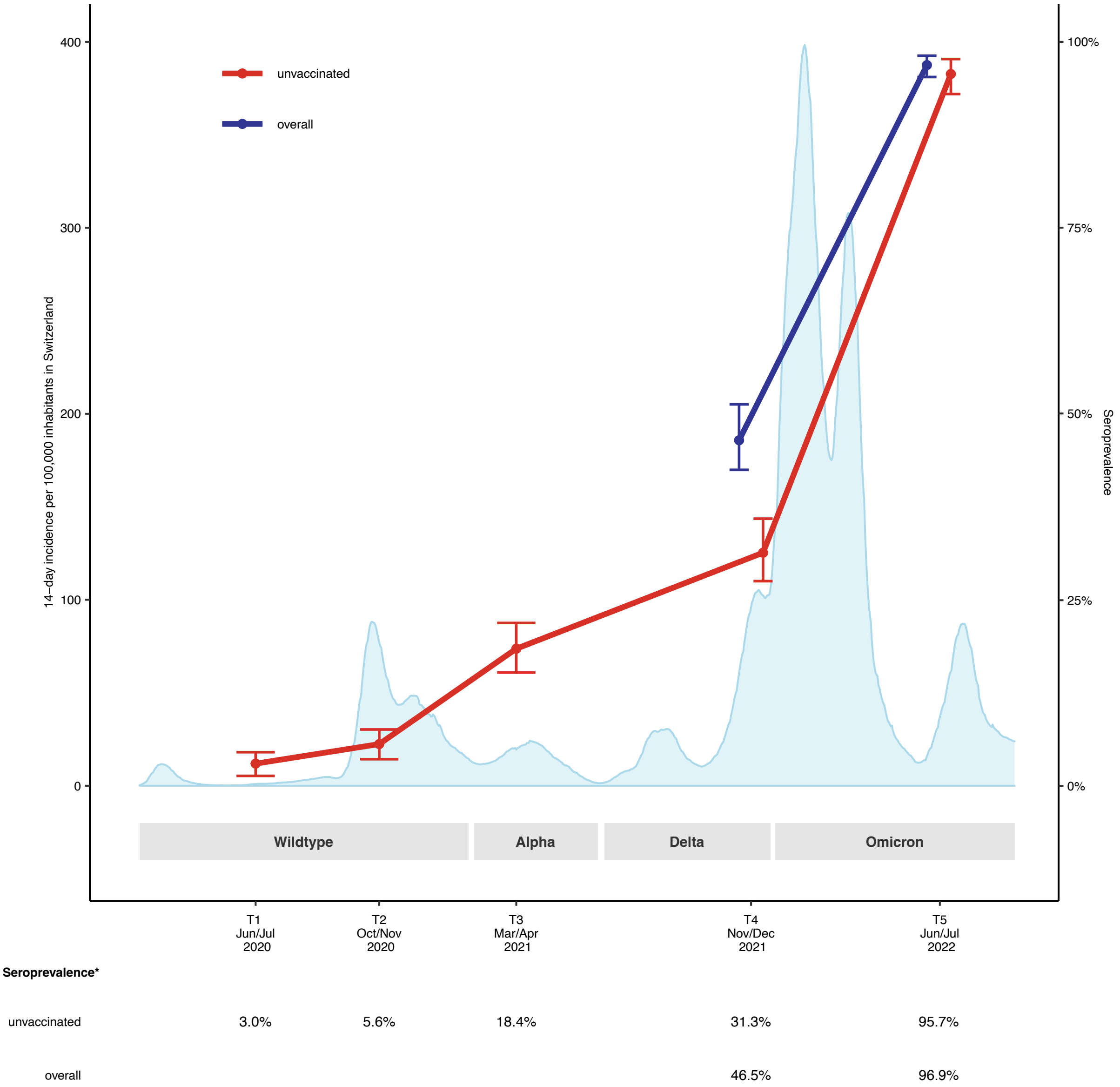

Supplementary Figure 2: Evolution of seroprevalence.

This figure shows the evolution of the incidence of diagnosed SARS-CoV-2 infections in Switzerland (light blue: the 14-day incidence per 100’000 inhabitants in Switzerland) and seroprevalence in children and adolescents from June 2020 to July 2022 in the cross-sectional cohort. Unvaccinated (red): unvaccinated children and adolescents across all five testing rounds (for the T1 to T3 it represents the overall population, since vaccination for children and adolescents was available starting from June 2021); overall (blue): all children and adolescents

participating (vaccination and/or infection); grey: predominant variant of concern in Switzerland (>50% of variants circulating). The error bars show 95% credible intervals. Source data are provided as a Source Data file.

*Seroprevalence was adjusted for school level, sex and district and test sensitivity and specificity.

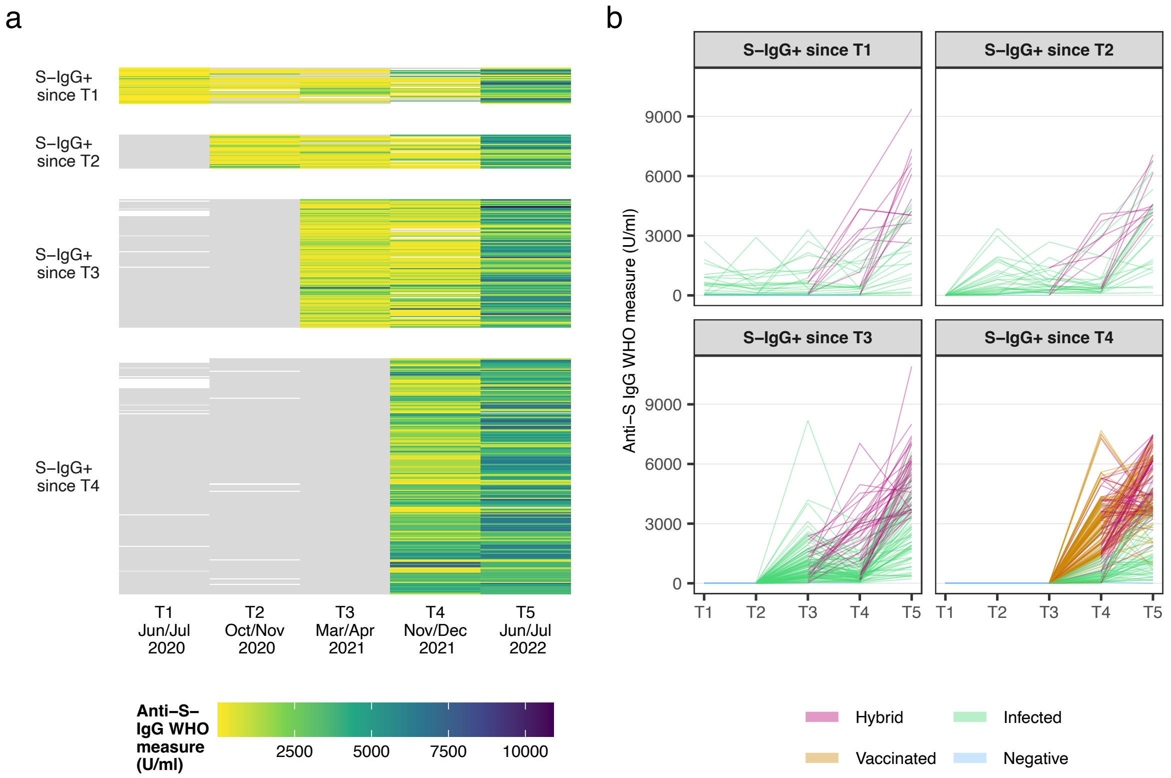

Supplementary Figure 3: Longitudinal trajectory of mean fluorescence intensity (MFI) ratio converted to WHO Units per millilitre (U/ml) in children and adolescents over the entire study period.

Individual trajectories of anti-spike IgG of mean fluorescence intensity (MFI) ratio converted to WHO units per millilitre (U/ml) over time separated by first incidence of seropositive result in children and adolescents (total n = 386 participants). S-IgG+: Anti-spike IgG positive WHO U/ml; 1) S-IgG+ since T1: n= 32 participants; 2) S-IgG+ since T2: n= 30 participants; 3) S-IgG+ since T3: n= 114 participants; 4) S-IgG+ since T4: n= 210 participants. Children and adolescents seroconverting from T4 to T5 are not shown (n= 328 participants). (**a**) The heatmap shows the changes in WHO U/ml through colour changes. Grey denotes seronegative anti-spike IgG result WHO U/ml. Colour denotes seropositive anti-spike IgG result with different WHO U/ml levels. White colour indicates no serology result available. 37 children and adolescent tested

seronegative throughout all five testing rounds are not shown in the figure. (**b**) This figure shows the individual trajectories of anti-spike IgG WHO U/ml levels, coloured by a child or adolescents' exposure status over time (i.e., hybrid (violet), vaccinated (orange), infected (green), negative (blue)). Negative denotes testing negative for anti-spike IgG; infected denotes unvaccinated individuals testing positive for anti-spike IgG; vaccinated denotes vaccinated individuals testing always negative for anti-spike IgG prior to vaccination and not testing positive for anti-nucleocapsid IgG; hybrid denotes individuals testing seropositive before getting vaccinated or were vaccinated and tested positive for anti-nucleocapsid-IgG antibodies. Source data are provided as a Source Data file.

T1: Jun/Jul 2020; T2: Oct/Nov 2020; T3: Mar/Apr 2021; T4: Nov/Dec 2021; T5: Jun/Jul 2022

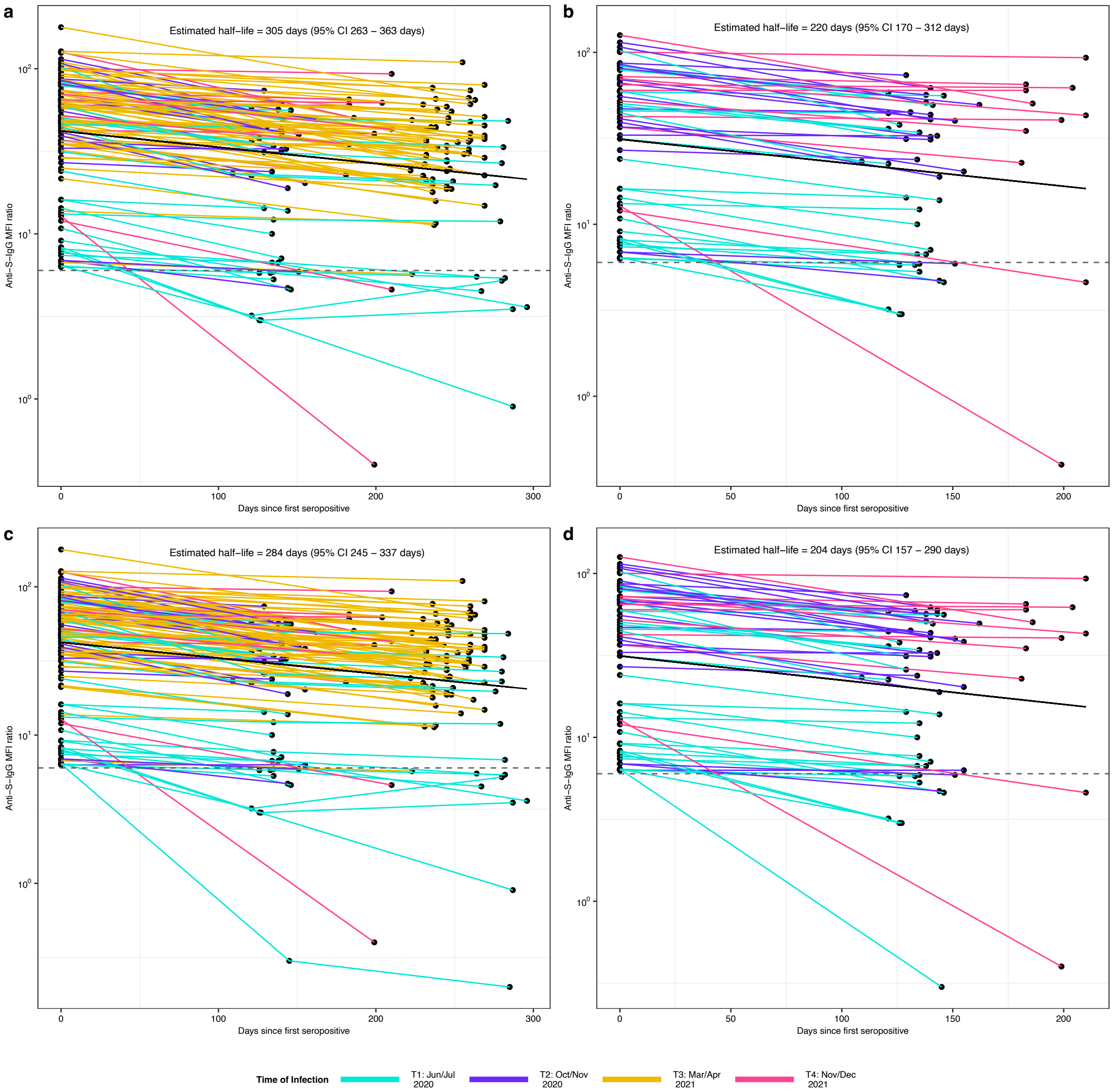

Supplementary Figure 4: Half-life of anti-spike IgG antibodies.

For the primary analysis, we calculated the waning of anti-spike IgG antibodies in children and adolescents considering a longer time frame of follow up of 365 days (n= 114 participants) **(a)** and a shorter time frame of 220 days (n= 53 participants) **(b)**. We followed up all children and adolescents from the timepoint of seroconversion. We excluded individuals who never tested seropositive for anti-spike IgG antibody, and who had no follow-up assessment after being tested seropositive and those who got vaccinated. A mixed linear model was conducted to

estimate the anti-spike IgG decay over time using a random intercept for participants. Each line presents the individual decay of anti-spike IgG antibody mean fluorescence intensity (MFI) ratio of a participant. The lines are coloured according to the timepoint of seroconversion, T1: Jun/Jul 2020 in light blue, T2: Oct/Nov 2020 in violet, T3: Mar/Apr 2021 in yellow and T4: Nov/Dec 2021 in pink.

Further, we performed a sensitivity analysis with a different longitudinal, which consisted of children and adolescents participating in four or more testing rounds, independent whether a T5 testing was done. When considering a time frame of follow up of 365 days (n= 131 participants) **(c)** and a time frame of 220 days (n= 60 participants) **(d)**. Source data are provided as a Source Data file.

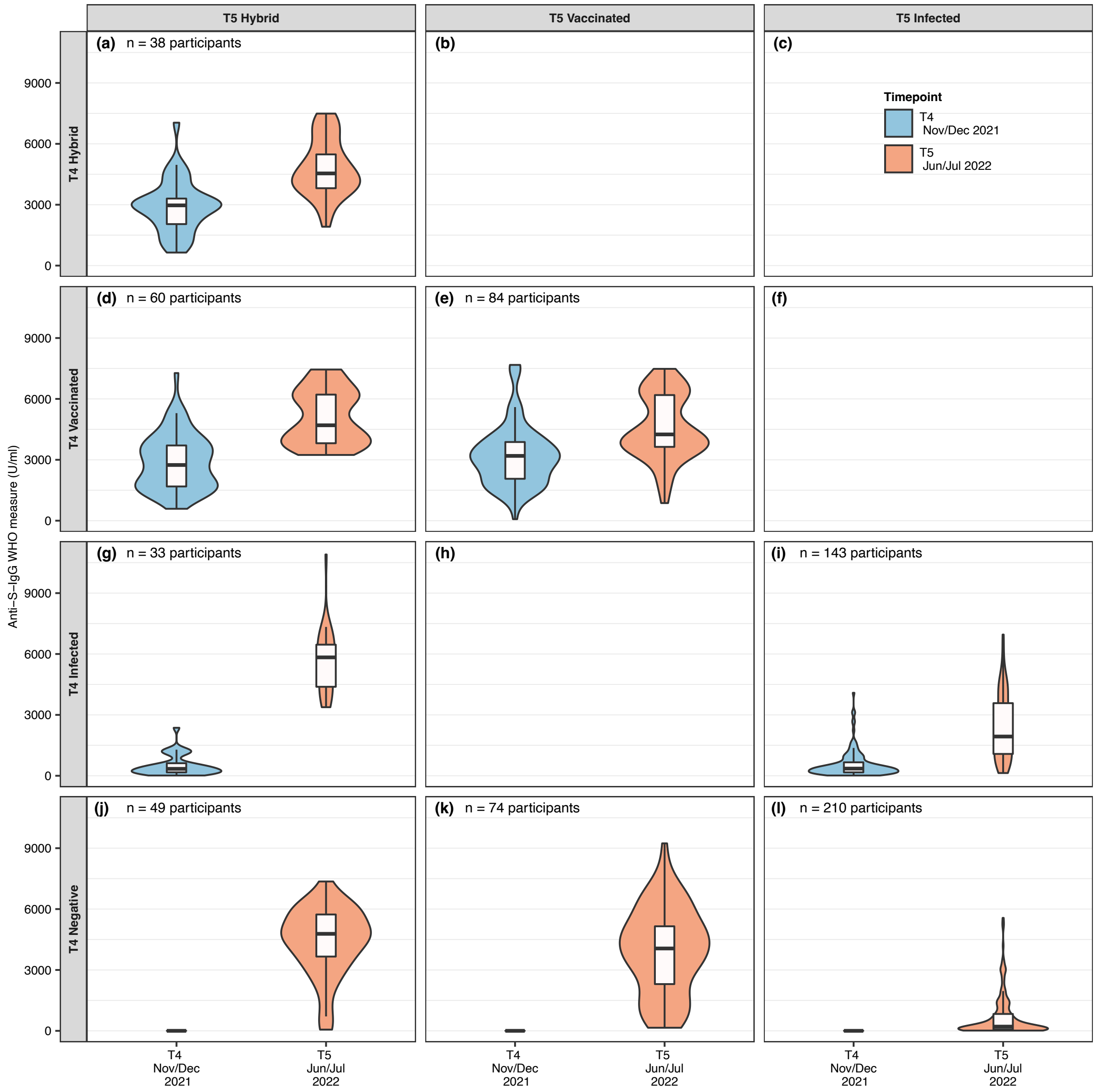

Supplementary Figure 5: Evolution of anti-spike IgG of mean fluorescence intensity (MFI) ratio converted to WHO Units per millilitre (U/ml) from T4 (Nov/Dec 2021) to T5 (Jun/Jul 2022).

This figure shows the evolution of anti-spike IgG antibodies in groups of children and adolescents separated by their serology and exposure status (i.e., negative, infected, vaccinated, or hybrid) at T4 (Nov/Dec 2021) and followed to T5 (Jun/Jul 2022). We converted the mean fluorescence intensity (MFI) values to WHO Units per millilitre (U/ml), by using the

Elecsys Anti-SARS-CoV2 immunoassay developed by Roche, for the purpose of interpretation (Supplementary Table 6). Panel (**a**) 38 participants with hybrid immunity at T4 and T5. Panel (**d**) 60 participants vaccinated at T4 and with hybrid immunity at T5. Panel (**e**) 84 participants vaccinated at T4 and T5. Panel (**g**) 33 participants infected at T4 and with hybrid immunity at T5. Panel (**i**) 143 participants infected at T4 and T5. Panel (**j**) 49 participants negative at T4 and with hybrid immunity at T5. Panel (**k**) 74 participants negative at T4 and vaccinated at T5. Panel (**l**) 210 participants negative at T4 and infected at T5. Negative denotes seronegative at T4; Infected denotes seropositive and unvaccinated (T4 infected denotes seropositive based on anti-spike IgG prior to the T4 testing and unvaccinated, T5 infected denotes seropositive based on anti-spike IgG between T4 and T5 and unvaccinated); Vaccinated denotes vaccinated participants but negative in previous rounds and without evidence for anti-nucleocapsid IgG response. Participants with hybrid immunity were seropositive before getting vaccinated, or were vaccinated and tested positive for anti-nucleocapsid-IgG antibodies. WHO U/ml levels at T4 and at T5 are shown in blue and orange, respectively. Boxplots in panels show the median and interquartile range (IQR; whisker: 1.5 IQR). 60 children and adolescents are not shown in the figure (n=31 participants were seronegative at T4 and T5, n=29 participants had no data at T4). Source data are provided as a Source Data file.

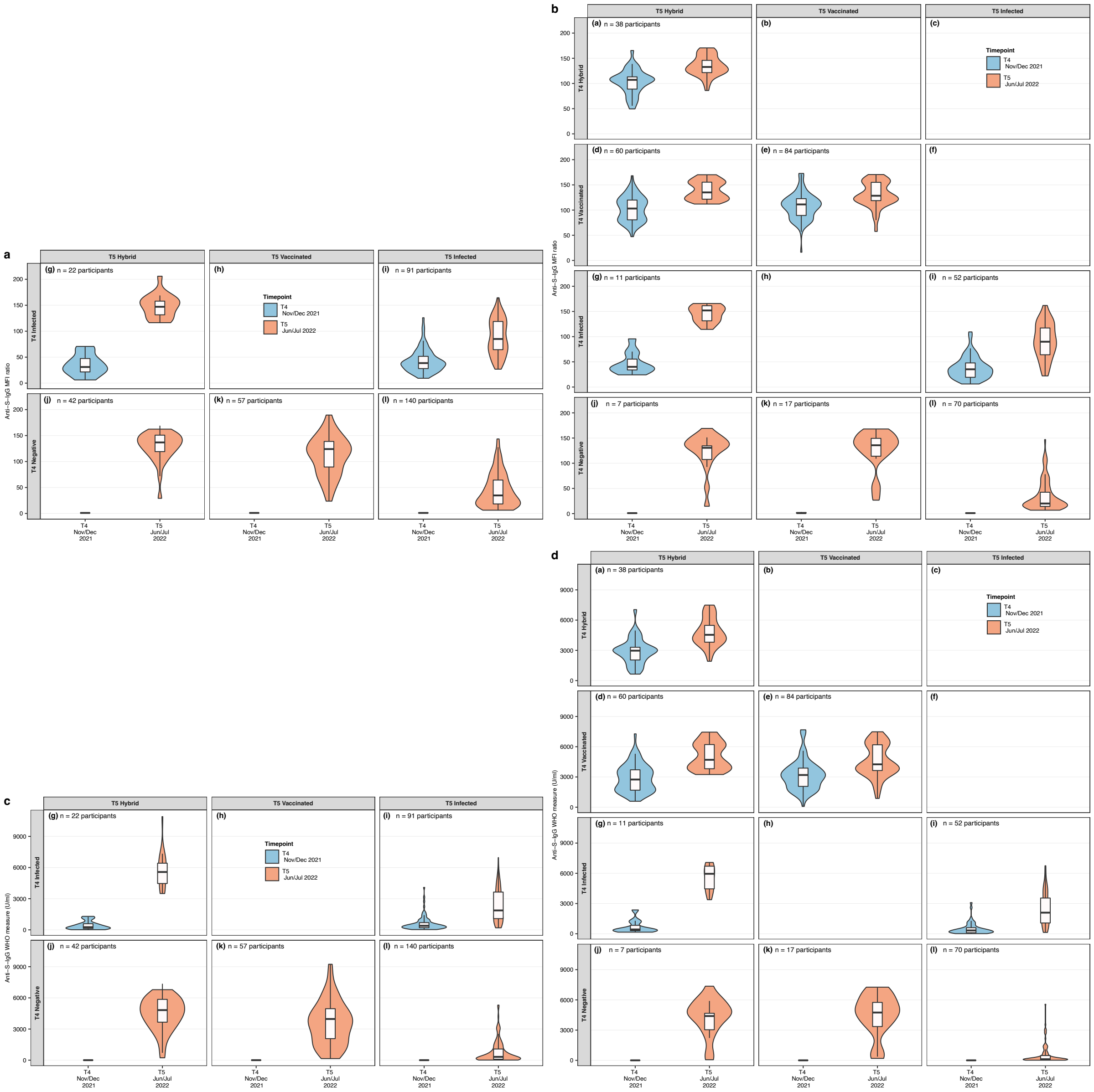

Supplementary Figure 6: Evolution of anti-spike IgG of mean fluorescence intensity (MFI) ratio stratified by age (<12 and ≥12 years age) from T4 (Nov/Dec 2021) to T5 (Jun/Jul 2022). This figure shows the evolution of anti-spike IgG antibodies stratified by age (<12 and ≥12 years of age) in participants separated by their serology and exposure status (i.e., negative, infected, vaccinated, or hybrid) at T4 (Nov/Dec 2021) and followed to T5 (Jun/Jul 2022) (Supplementary Table 7). Figure **(a)** shows children younger than 12 years and Figure **(b)**

shows adolescents 12 years or older with mean fluorescence intensity (MFI) values. Figure **(c)** shows children younger than 12 years Figure **(d)** shows adolescents 12 years and older and with MFI values converted to WHO Units per millilitre (U/ml). Panel (**a**) shows participants with hybrid immunity at T4 and T5. Panel (**d**) shows participants vaccinated at T4 and with hybrid immunity at T5. Panel (**e**) shows participants vaccinated at T4 and T5. Panel (**g**) shows participants infected at T4 and with hybrid immunity at T5. Panel (**i**) shows participants infected at T4 and T5. Panel (**j**) shows participants negative at T4 and with hybrid immunity at T5. Panel (**k**) shows participants negative at T4 and vaccinated at T5. Panel (**l**) shows participants negative at T4 and infected at T5. Negative denotes seronegative at T4; Infected denotes seropositive and unvaccinated (T4 infected denotes seropositive based on anti-spike IgG prior to the T4 testing and unvaccinated, T5 infected denotes seropositive based on anti-spike IgG between T4 and T5 and unvaccinated); Vaccinated denotes vaccinated participants but negative in previous rounds and without evidence for anti-nucleocapsid IgG response. Participants with hybrid immunity were seropositive before getting vaccinated, or were vaccinated and tested positive for anti-nucleocapsid-IgG antibodies. Anti-spike IgG antibody levels at T4 and at T5 are shown in blue and orange, respectively. Boxplots in panels show the median and interquartile range (IQR; whisker: 1.5 IQR). 60 children and adolescents are not shown in the figure (n=31 participants were seronegative at T4 and T5, n=29 participants had no data at T4). Source data are provided as a Source Data file.

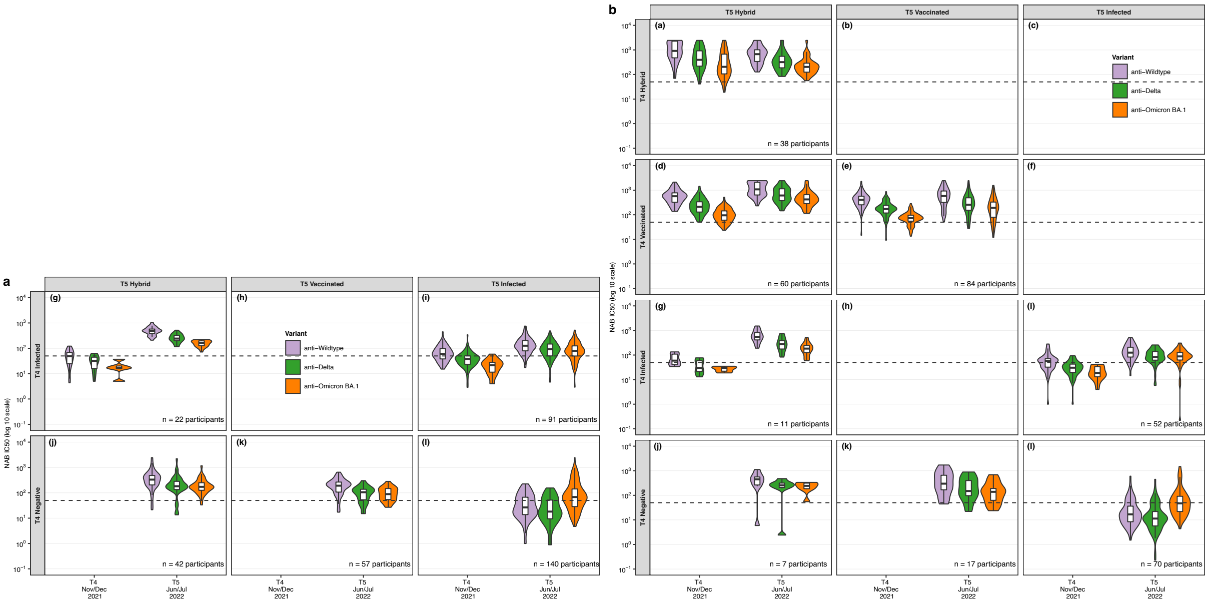

Supplementary Figure 7: Evolution of neutralizing antibodies stratified by age (<12 and ≥12 years age) between T4 (Nov/Dec 2021) to T5 (Jun/Jul 2022).

This figure shows the development of neutralising antibodies against different SARS-CoV-2 variants between T4 (Nov/Dec 2021) and T5 (Jun/Jul 2022) stratified by age (<12 and ≥12 years of age). Figure **(a)** shows the children <12 years and Figure **(b)** shows the adolescents ≥12 years of age. Children and adolescents are categorised based on their exposure status (i.e., hybrid, vaccinated, infected, negative). Panel (**a**) shows participants with hybrid immunity at T4 and T5. Panel (**d**) shows participants vaccinated at T4 and with hybrid immunity at T5. Panel (**e**) shows participants vaccinated at T4 and T5. Panel (**g**) shows participants infected at T4 and with hybrid immunity at T5. Panel (**i**) shows participants infected at T4 and T5. Panel (**j**) shows participants negative at T4 and with hybrid immunity at T5. Panel (**k**) shows participants negative at T4 and vaccinated at T5. Panel (**l**) shows participants negative at T4 and infected at T5. Negative denotes seronegative at T4; Infected denotes seropositive and unvaccinated (T4 infected denotes seropositive based on anti-spike IgG prior to the T4 testing and

unvaccinated, T5 infected denotes seropositive based on anti-spike IgG between T4 and T5 and unvaccinated); Vaccinated denotes vaccinated participants but negative in previous rounds and without evidence for anti-nucleocapsid IgG response. Children and adolescents with hybrid immunity were seropositive before getting vaccinated, or were vaccinated and tested positive for anti-nucleocapsid-IgG antibodies. Dotted line indicates NAB IC50 value threshold (50) for neutralising activity. Children and adolescents with NAB IC50 values above the threshold are assumed to have 50% or higher neutralisation capacity. Boxplots in panels show the median and interquartile range (IQR; whisker: 1.5 IQR). 60 children and adolescents are not shown in the figure (n=31 participants were seronegative at T4 and T5, n=29 participants had no data at T4). Source data are provided as a Source Data file.
