# Supplementary material for: Persistent humoral immune response in youth throughout the COVID-19 pandemic: prospective school-based cohort study
Source: Nat Commun. 2023 Nov 27;14:7764. doi: 10.1038/s41467-023-43330-y (PMC10682435; doi:10.1038/s41467-023-43330-y)
Supplement: Supplementary file 3 — Description of Additional Supplementary Files [file 41467_2023_43330_MOESM3_ESM.pdf]

Description of Additional Supplementary Files

File Name: Supplementary Software
Description: R code to estimate seroprevalence, and create tables and figures. STAN model used to estimate seroprevalence.
