# Supplementary material for: Persistent humoral immune response in youth throughout the COVID-19 pandemic: prospective school-based cohort study
Source: Nat Commun. 2023 Nov 27;14:7764. doi: 10.1038/s41467-023-43330-y (PMC10682435; doi:10.1038/s41467-023-43330-y)
Supplement: Supplementary file 5 — Reporting Summary [file 41467_2023_43330_MOESM5_ESM.pdf]

Corresponding author(s): Prof. Dr. med Susi Kriemler

Last updated by author(s): October 26, 2023

Reporting Summary

Nature Portfolio wishes to improve the reproducibility of the work that we publish. This form provides structure for consistency and transparency in reporting. For further information on Nature Portfolio policies, see our Editorial Policies and the Editorial Policy Checklist.

Statistics

For all statistical analyses, confirm that the following items are present in the figure legend, table legend, main text, or Methods section.

n/a	Confirmed	
☐	☒	The exact sample size (*n*) for each experimental group/condition, given as a discrete number and unit of measurement
☐	☒	A statement on whether measurements were taken from distinct samples or whether the same sample was measured repeatedly
☐	☒	The statistical test(s) used AND whether they are one- or two-sided<br>*Only common tests should be described solely by name; describe more complex techniques in the Methods section.*
☐	☒	A description of all covariates tested
☐	☒	A description of any assumptions or corrections, such as tests of normality and adjustment for multiple comparisons
☐	☒	A full description of the statistical parameters including central tendency (e.g. means) or other basic estimates (e.g. regression coefficient) AND variation (e.g. standard deviation) or associated estimates of uncertainty (e.g. confidence intervals)
☐	☒	For null hypothesis testing, the test statistic (e.g. *F*, *t*, *r*) with confidence intervals, effect sizes, degrees of freedom and *P* value noted<br>*Give P values as exact values whenever suitable.*
☐	☒	For Bayesian analysis, information on the choice of priors and Markov chain Monte Carlo settings
☒	☐	For hierarchical and complex designs, identification of the appropriate level for tests and full reporting of outcomes
☐	☒	Estimates of effect sizes (e.g. Cohen's *d*, Pearson's *r*), indicating how they were calculated

Our web collection on statistics for biologists contains articles on many of the points above.

Software and code

Policy information about availability of computer code

Data collection	Immune assay data:<br>Luminex analysis of Spike-specific IgG and Nucleocapsid-specific IgG and SARS-CoV-2 Spike neutralization activity analysis: Samples were read on a Bio-Plex (Luminex) 200 plate reader with Bio-Plex Manager software (version 6.2; Bio-Rad) to obtain a mean fluorescence intensity (MFI) value for each sample.<br><br>Electronic survey data:<br>The Research Electronic Data Capture (REDCap) platform was used for data collection (current version 13.4.13) for all electronic survey data. REDCap is a publicly available, web-based application created and distributed by Vanderbilt University.
Data analysis	Statistical analyses:<br>All statistical analyses were performed using R (v4.2.1), using the tidyverse (v1.3.2), lmerTest (v3.1-3), epitools (v0.5-10.1), lubridate (v1.8.0), janitor (v2.1.0), openxlsx (v4.2.5), broom.mixed (v0.2.9.4) packages, including the RSTAN package (v2.26.16) to fit the Bayesian models. Results were visualized using the ggplot2 (v3.3.6), scales (v1.2.1), cowplot(1.1.1) and RColorBrewer (v1.1-2) packages.

For manuscripts utilizing custom algorithms or software that are central to the research but not yet described in published literature, software must be made available to editors and reviewers. We strongly encourage code deposition in a community repository (e.g. GitHub). See the Nature Portfolio guidelines for submitting code & software for further information.

Data

Policy information about availability of data

All manuscripts must include a data availability statement. This statement should provide the following information, where applicable:

- Accession codes, unique identifiers, or web links for publicly available datasets
- A description of any restrictions on data availability
- For clinical datasets or third party data, please ensure that the statement adheres to our policy

All data supporting the findings of this study are available within the paper and its supplementary information files.
Source data are provided with this paper (see Source Data file).
The analysis code used for this study (R programming language) can be found in the Supplementary Software file.

Research involving human participants, their data, or biological material

Policy information about studies with human participants or human data. See also policy information about sex, gender (identity/presentation), and sexual orientation and race, ethnicity and racism.

Reporting on sex and gender	We used the term sex (biological attribute) when collecting data in the questionnaires. Sex was determined based on self-reporting. The distribution of sex in our study is reported in Table 1.
Reporting on race, ethnicity, or other socially relevant groupings	We did collect information on race or ethnicity. Reporting on race, ethnicity or other socially relevant group is not applicable.
Population characteristics	The study population characteristics are reported in detail in Table 1. We tested between 1876 and 2500 children and andolescents at each testing round between June 2020 and July 2022 and 751 children and adolescents were included in the longitudinal cohort. Median age (interquartle range) is as follows: round 1: 12 (6-17), round 2 12: (7-17), round 3: 12 (7-17), round 4: 12 (7-17), and round 5: 12 (7-18). Number and proportion of male participants: round 1: 1197/2473 (48%), round 2: 1211/2500 (48%), round 3: 1165/2450 (48%), round 4: 884/1875 (47%), and round 5: 990/2105 (47%). None of the participants in rounds 1 to 3 were vaccinated. 476/1876 (25%) participants in round 4 and 913/2105 (43%) in round 5 were vaccinated.
Recruitment	One out of six (1.5 million) inhabitants of Switzerland lives in the canton of Zurich. The canton is divided into 12 districts. Primary school is divided into lower level with grades 1–3 (kindergarten not included in this study) and middle level with grades 4–6. Secondary school comprises upper level with grades 7–9. A significant proportion of schools (around 10% of schools), particularly in rural setting, adopt age-mixed learning methodology, in which students of two or three adjacent grades are taught in the same classroom.<br>This repeated cross-sectional analysis is based on a prospective cohort study, using data from children and adolescents from randomly selected schools and classes in the canton of Zurich, Switzerland. We stratified the random selection of schools within districts of the canton, and random selection of classes was stratified within lower, middle, and upper levels of schools. All children attending the selected classes were invited, except in mixed-age classes (in which only students from the eligible grades were invited). We selected the primary schools randomly and matched the closest secondary school geographically. The targeted number of schools to enroll per district ranged from 2 to 10, depending on the district population size. After the initial invitation round, overall school participation rate was assessed and additional schools were selected within required districts, until we reached the aimed number, or further recruitment was not feasible. Full details on the recruitment process are reported in the study protocol (https://doi.org/10.1007/s00038-020-01495-z) and in the method section of the manuscript.<br>On school-level no selection bias was observed. The socioeconomic context of the region (in the canton of Zurich) did not differ for non-participating and participating schools, and it was not associated with participation rates within schools (https://doi.org/10.1136/bmj.n616). However, selection bias could have happend on the participant-level, due to the high socioeconomic status of participants than in the general population. During the Omicron period, participation bias was not a concern, as all children and adolescents were inevitably exposed to an infection.
Ethics oversight	The study was approved by the Cantonal Ethics Committee Zurich (2020-01336).

Note that full information on the approval of the study protocol must also be provided in the manuscript.

Field-specific reporting

Please select the one below that is the best fit for your research. If you are not sure, read the appropriate sections before making your selection.

☒ Life sciences ☐ Behavioural & social sciences ☐ Ecological, evolutionary & environmental sciences

For a reference copy of the document with all sections, see nature.com/documents/nr-reporting-summary-flat.pdf

Life sciences study design

All studies must disclose on these points even when the disclosure is negative.

Sample size	We stratified the random selection of schools within districts of the canton, and random selection of classes was stratified within lower, middle, and upper levels of schools. All children attending the selected classes are invited, except in mixed-age classes (in which only students from the eligible grades are invited).<br>We selected the primary schools randomly and matched the closest secondary school geographically. The targeted number of schools to enroll per district ranged from 2 to 10, depending on the district population size. After the initial invitation round, overall school participation rate was assessed and additional schools were selected within required districts, until we reached the aimed number, or further recruitment was not feasible. Out of 55 randomly selected school we cross-sectionally included between 1876 an 2500 children and adolescents. In the longitudinal cohort we included 751 children and adolescents, who participated in four out of five testing rounds. Full details on the sample size calculation are reported in the study protocol (https://doi.org/10.1007/s00038-020-01495-z).
Data exclusions	Exclusion criteria were pre-established and were as follows: for children – attendance of kindergarten grades (due to difficulties in enrolling, collecting venous blood samples, and following up the cohort); for all participants – any acute respiratory infection with symptoms present within the last 48 hours before the testing at school; and severe acute respiratory syndrome coronavirus 2 (SARS-CoV-2) infection confirmed with reverse transcription polymerase chain reaction (RT-PCR) and symptom onset within the last 21 days prior to testing; and no written consent by a parent or caregiver.<br>Exclusion criteria for the estimation of anti-spike antibody IgG half-life were: For these half-life analyses we only included infected (based on exposure status (i.e., hybrid, vaccinated, infected, negative)) children and adolescents. We excluded all children and adolescents with a potential reinfection, defined as the presence of a newly positive anti-nucleocapsid IgG antibody or any increase in anti-spike IgG titres between two testing points. More details can be found in the manuscript.<br>Exclusion criteria for the quantification of reinfections in children and adolescents were: To quantify the proportions of children and adolescents with prior infection, vaccination, or both at T4 who experienced an infection or reinfection between T4 and T5, we used changes in antibody titres to determine (re-)infection events due to the high number of undiagnosed infections during the Omicron wave. We only included children and adolescents who were seropositive due to infection, vaccination, or both at T4 and excluded all other children and adolescents.
Replication	Due to limited availability of biological samples, the majority of samples were tested only once in antibody and neutralizing antibody analyses. All performed tests were validated prior to testing of the samples from this study.
Randomization	No randomization was performed due to the observational nature of this study.
Blinding	No group allocation was performed due to the observational nature of this study. Investigators performing antibody and neutralizing antibody testing did not have knowledge of the clinical characteristics of participants corresponding to the samples at the time of testing.

Reporting for specific materials, systems and methods

We require information from authors about some types of materials, experimental systems and methods used in many studies. Here, indicate whether each material, system or method listed is relevant to your study. If you are not sure if a list item applies to your research, read the appropriate section before selecting a response.

Materials & experimental systems

n/a	Involved in the study	
☐	☒	Antibodies
☒	☐	Eukaryotic cell lines
☒	☐	Palaeontology and archaeology
☒	☐	Animals and other organisms
☐	☒	Clinical data
☒	☐	Dual use research of concern
☒	☐	Plants

Methods

n/a	Involved in the study	
☒	☐	ChIP-seq
☒	☐	Flow cytometry
☒	☐	MRI-based neuroimaging

Antibodies

Antibodies used	Secondary antibodies for Luminex analysis of Spike-Specific IgG and Nucleocapsid-Specific IgG: Polyclonal Goat F(ab')2 anti-human IgA-PE (Southern Biotech, Catalog 2052-09, Dilution 1:100); Polyclonal Goat anti-human IgG-PE (OneLambda, Catalog LS-AB2, Dilution 1:100).<br><br>Secondary antibodies for Luminex analysis of SARS-CoV-2 Spike neutralization activity: Polyclonal Goat F(ab') anti-mouse IgG (Invitrogen, Catalog 12-4010-87, Dilution 1:100).<br><br>Positive control for 100% neutralization consist of high concentration (>1ug/ml) of two broadly neutralizing human monoclonal antibodies binding distinct epitopes on the SARS-CoV-2 Spike protein, isolated from previously infected and vaccinated donors, reported in: Fenwick et al. A highly potent antibody effective against SARS-CoV-2 variants of concern. Cell Reports. 2021. https//doi.org/10.1016/j.celrep.2021.109814. Fenwick et al. Patient-derived monoclonal antibody neutralizes SARS-CoV-2 Omicron variants

and confers full protection in monkeys. Nature Microbiology. 2022. https://doi.org/10.1038/s41564-022-01198-6.

Validation	Antibody testing for anti-S-IgG and anti-N-IgG using the Luminex-based assay was validated prior to use in this study and is reported in: Fenwick et al. Changes in SARS-CoV-2 Spike versus Nucleoprotein Antibody Responses Impact the Estimates of Infections in Population-Based Seroprevalence Studies. J. Virol. 2021. https://www.doi.org/10.1128/JVI.01828-20.<br><br>In another study by our group the Luminex assay was validated against Roche (Roche Elecsys Anti-SARS-CoV-2). Menges, D., Zens, K.D., Ballouz, T. et al. Heterogenous humoral and cellular immune responses with distinct trajectories post-SARS-CoV-2 infection in a population-based cohort. Nat Commun 13, 4855 (2022). https://doi.org/10.1038/s41467-022-32573-w<br><br>Neutralization assays were validated prior to use in this study and is reported in: Fenwick et al. A high-throughput cell- and virus-free assay shows reduced neutralization of SARS-CoV-2 variants by COVID-19 convalescent plasma. Science Translational Medicine. 2021. https://www.doi.org/10.1126/scitranslmed.abi8452. Cross-validation for the Spike-ACE2 surrogate neutralization assay developed in these studies used serum samples from a panel of 206 SARS-CoV-2 seropositive individuals with varying degrees of infection severity in parallel with a conventional authentic live virus cytopathic effect (CPE) assay in Vero cells. Of note, serum samples from 27 children were tested in these validation studies and gave the same highly correlative neutralizing antibody response in the two assays compared to the adult serum samples. Positive control antibodies for 100% neutralization were equally validated prior to use and reported in: Fenwick et al. A highly potent antibody effective against SARS-CoV-2 variants of concern. Cell Reports. 2021. https//doi.org/10.1016/j.celrep.2021.109814. Patient-derived monoclonal antibody neutralizes SARS-CoV-2 Omicron variants and confers full protection in monkeys. Nature Microbiology. 2022. https://doi.org/10.1038/s41564-022-01198-6.

Clinical data

Policy information about clinical studies

All manuscripts should comply with the ICMJE guidelines for publication of clinical research and a completed CONSORT checklist must be included with all submissions.

Clinical trial registration	This observational cohort study was prospectively registered on clinicaltrials.gov (NCT04448717).
Study protocol	The study protocol has been published: https://doi.org/10.1007/s00038-020-01495-z
Data collection	In all five testing rounds, venous blood samples were collected at the individual schools in the canton of Zurich. The first testing round (T1) was performed in June/July 2020, the second (T2) in October/November 2020, the third (T3) in March/April 2021, the fourth (T4) in November/December 2021 and the last fifth (T5) testing round in June/July 2022. As shown in the study participant flow chart (Figure 1), we followed corresponding repeated cross-sectional cohorts and a longitudinal cohort. The longitudinal cohort consisted of children and adolescents participating in the last (T5) and at least three previous testing rounds.<br>Online questionnaires were sent to participants at enrolment and repeatedly every 3 to 6 months over the duration of the study, collecting information on sociodemographic characteristics, chronic conditions, and vaccination status.
Outcomes	The primary outcomes were the longitudinal development of the anti-spike IgG antibodies and neutralising antibody responses against SARS-CoV-2 in school aged children and adolescents over time. The secondary outcomes were the persistence of antibodies and variation of antibody levels in individuals only infected, vaccinated or with hybrid immunity during the early Omicron period.
